# Reduction and Functional Exhaustion of T Cells in Patients With Coronavirus Disease 2019 (COVID-19)

**DOI:** 10.3389/fimmu.2020.00827

**Published:** 2020-05-01

**Authors:** Bo Diao, Chenhui Wang, Yingjun Tan, Xiewan Chen, Ying Liu, Lifen Ning, Li Chen, Min Li, Yueping Liu, Gang Wang, Zilin Yuan, Zeqing Feng, Yi Zhang, Yuzhang Wu, Yongwen Chen

**Affiliations:** ^1^Department of Medical Laboratory Center, General Hospital of Central Theater Command, Wuhan, China; ^2^Institute of Immunology, PLA, Third Military Medical University, Chongqing, China; ^3^Medical English Department, College of Basic Medical Sciences, Army Medical University, Chongqing, China; ^4^Department of Medical Laboratory Medicine, General Hospital of Central Theater Command, Wuhan, China; ^5^Hanyang Hospital Affiliated to Wuhan University of Science and Technology, Wuhan, China

**Keywords:** SARS- CoV-2, COVID-19, T cell reduction, T cell exhaustion, cytokine strom

## Abstract

**Background:** The outbreak of coronavirus disease 2019 (COVID-19) caused by severe acute respiratory syndrome coronavirus 2 (SARS-CoV-2) has posed great threat to human health. T cells play a critical role in antiviral immunity but their numbers and functional state in COVID-19 patients remain largely unclear.

**Methods:** We retrospectively reviewed the counts of T cells and serum cytokine concentration from data of 522 patients with laboratory-confirmed COVID-19 and 40 healthy controls. In addition, the expression of T cell exhaustion markers were measured in 14 COVID-19 cases.

**Results:** The number of total T cells, CD4^+^ and CD8^+^ T cells were dramatically reduced in COVID-19 patients, especially in patients requiring Intensive Care Unit (ICU) care. Counts of total T cells, CD8^+^ T cells or CD4^+^ T cells lower than 800, 300, or 400/μL, respectively, were negatively correlated with patient survival. T cell numbers were negatively correlated to serum IL-6, IL-10, and TNF-α concentration, with patients in the disease resolution period showing reduced IL-6, IL-10, and TNF-α concentrations and restored T cell counts. T cells from COVID-19 patients had significantly higher levels of the exhausted marker PD-1. Increasing PD-1 and Tim-3 expression on T cells was seen as patients progressed from prodromal to overtly symptomatic stages.

**Conclusions:** T cell counts are reduced significantly in COVID-19 patients, and the surviving T cells appear functionally exhausted. Non-ICU patients with total T cells counts lower than 800/μL may still require urgent intervention, even in the immediate absence of more severe symptoms due to a high risk for further deterioration in condition.

## Introduction

In December 2019, a series of acute respiratory illnesses were reported in Wuhan, Hubei Province, China (1, 2). A novel coronavirus, initially named severe acute respiratory syndrome coronavirus 2 (SARS-CoV-2), was identified as the cause of this disease by the Chinese Center for Disease Control and Prevention (CDC) (3). This disease, now designated as coronavirus disease 2019 (COVID-19) by the WHO, rapidly spread to other cities of China, and has become a public health emergency of international concern (PHEIC) following its global spread. COVID-19 clinically manifests as fever, cough, muscle pain, fatigue, diarrhea and pneumonia, and can cause death in severe cases (4–6). Up through March 20, 2020, China has reported 81008 cases of confirmed COVID-19 and 3,255 fatalities (7).

Since an effective immune response against viral infections depends on the activation of cytotoxic T cells that can clear infection by killing virus-infected cells (8), boosting the numbers and function of T cells in COVID-19 patients is critical for successful recovery. A recent study reported that the 82.1% of COVID-19 cases displayed low circulating lymphocyte counts (4–6). However, the factors which might cause the reduction in count, and the activation status of T cells in COVID-19 patients, remain uninvestigated. We retrospectively analyze here the clinical data from 522 cases of COVID-19 who were admitted into the General Hospital of Central Theater Command and Hanyang Hospital in Wuhan from December 2019 to January 2020. We also compare the expression of exhaustion markers PD-1 and Tim-3 on the surface of CD4^+^ and CD8^+^ T cells from COVID-19 patients and healthy controls. Our results thus provide a preliminary demonstration of T cell exhaustion during COVID-19 infection and suggest that more urgent, early intervention may be required in patients with low T lymphocyte counts.

## Methods

### Patients

Medical records from 522 patients (aged from 5 days to 97 years) with confirmed COVID-19 and admitted into the General Hospital of Central Theater Command or Hanyang Hospital in Wuhan from December 2019 to January 2020, and 40 healthy people (aged from 2 to 62 years), who came to the hospitals for routine physical examination, were collected and retrospectively analyzed. Diagnosis of COVID-19 was based on the New Coronavirus Pneumonia Prevention and Control Program (5th edition) published by the National Health Commission of China (9). All the patients were laboratory-confirmed positive for SARS-CoV-2 by use of quantitative RT-PCR (qRT-PCR) of throat swab samples. This study was approved by the National Health Commission of China and Ethics Commission of General Hospital of Central Theater Command ([2020]-004-1) and Hanyang Hospital (20200217). Written informed consent was waived by the Ethics Commission of the designated hospital for emerging infectious diseases.

### Definitions

The classification of clinical types, which consist of mild/moderate/severe/critical, was based on the New Coronavirus Pneumonia Prevention and Control Program (5th edition) published by the National Health Commission of China (9). Within the analyzed cohort, 43 patients were admitted to the intensive care unit (ICU), because they required high-flow nasal cannula or higher-level oxygen support measures to correct hypoxaemia. Hypoxaemia was defined as arterial oxygen tension (PaO_2_) over inspiratory oxygen fraction (FIO_2_) of <300 mm Hg or arterial oxygen saturation of 93% or lower. According to the staging of infectious disease (10), the prodromal period is a phase in which the host begins to experience general signs and symptoms. The illness period (overtly symptomatic period) is a phase in which the signs or symptoms of disease are most obvious and severe, with positive laboratory findings and chest/lung pathological manifestations. For ICU patients, ICU period is a phase in which the symptoms are most obvious and severe. The decline period is a phase in which the clinical symptoms begin to decline, laboratory findings and chest pathological signs improve, and arterial oxygen saturation normalizes.

### Data Collection

We reviewed clinical records, nursing records, laboratory findings, and chest X-rays or CT scans for all the patients and physical examination records of the 40 healthy people. All information was obtained and curated with a customized data collection form. Three investigators (C Wang, Z Fen, and Y Chen) independently reviewed the data collection forms to verify data accuracy.

### Sample Collection and Flow Cytometric Analysis

Peripheral blood samples from 14 patients and 3 healthy volunteers were simultaneously processed in the Central Lab of General Hospital of Central Theater Command to isolate peripheral blood mononuclear cells (PBMCs) for further testing. The peripheral blood was supplemented with anticoagulants (EDTA-K_2_) and PBMCs were harvested by density gradient centrifugation. Isolated PBMCs were stained with a BD multitest IMK Kit (Cat340503, BD Biosciences) to analyze the frequency and cell number of total, CD4^+^ and CD8^+^ T cells, as well as B and NK cells in healthy controls and patients. The exhaustion of T cells was detected using human CD4-PerCP (RPA-T4, Biolegend), CD8-APC (SK1, BD Biosciences), CD8-PE (SK1, Biolegend), PD-1-PE (EH12.2H7, Biolegend), and TIM-3-FITC (F38-2E2, Biolegend) antibodies. After being stained, the cells were measured by flow cytometry on an LSR Fortessa Cell Analyzer (BD Biosciences) and data analyzed using the FlowJo software (TreeStar). All experimental procedures were completed under biosafety level II plus condition.

### Statistical Analysis

**Figure 1:**
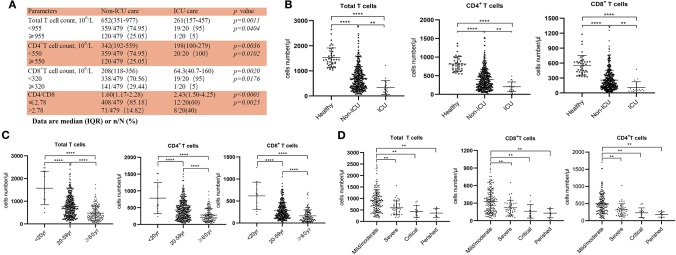
Reduced T cell numbers in COVID-19 patients. **(A)** Demographics of T cells in patients; **(B)** T cell numbers in different groups; **(C)** T cell numbers in patients of different ages; **(D)** T cell count in Non-ICU care patients with different clinical outcomes. **p < 0.01 and ****p < 0.0001.

**Figure 2:**
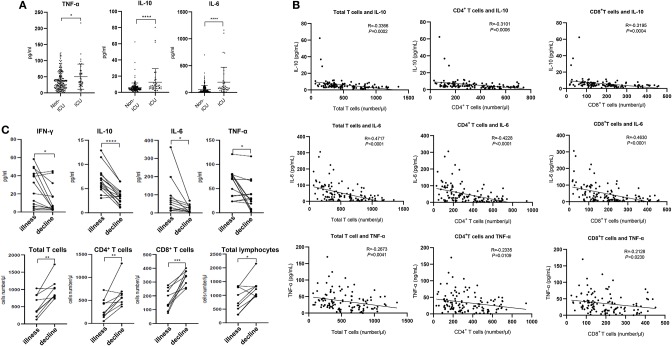
Cytokines and relative T cell numbers in COVID-19 patients. **(A)** Cytokine levels in different groups; **(B)** Relationship between T cell numbers and cytokine levels; **(C)** Dynamic profiles of cytokine levels and T cell numbers in Non-ICU care patients. *p < 0.05, **p < 0.01, ***p < 0.001, and ****p < 0.0001.

**Figure 3:**
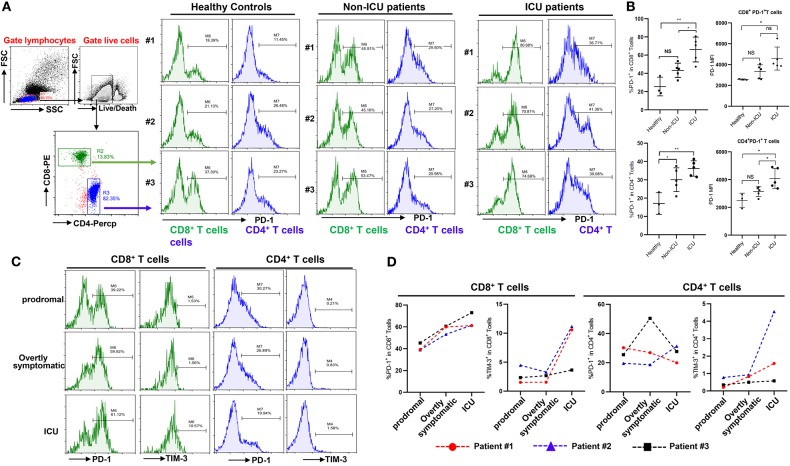
Exhaustion of T cells in COVID-19 patients. **(A,B)** PD-1 expression on T cells in different groups; **(C,D)** Dynamic profile of PD-1 and TIM-3 expression on T cells in 3 patients. NS: not significant, *p < 0.05, **p < 0.01.

Statistical analyses were performed using GraphPad Prism version 8.0 (GraphPad Software, Inc., San Diego, CA, USA). Continuous variables were directly expressed as a range. Categorical variables were expressed as numbers/NUMBERS (%). Data in **Figure 2B** are analyzed using linear regression and R values are from Pearson's correlation coefficient test. *p*-values are from χ^2^ (Figure 1A), non-paired *t*-test (Figures 1A, 2A, Supplementary Figure 1A), paired *t*-test (Figure 2C, Supplementary Figure 1B), ordinary one-way ANOVA (Figures 1B–D, 3B,D, Supplementary Figure 1C) and Pearson's correlation coefficient *t*-test (Figure 2B).

### Role of the Funding Source

The funding agencies did not participate in study design, data collection, data analysis, or manuscript writing. The corresponding authors were responsible for all aspects of the study to ensure that issues related to the accuracy or integrity of any part of the work were properly investigated and resolved. The final version was approved by all authors.

## Results

### Decreased Numbers of Total T Cells, CD4^+^, and CD8^+^ Subsets in COVID-19 Patients

From our retrospective analysis of 522 patients, 499 cases had lymphocyte count recorded. 75.75% (359/499), 75.95% (379/499), and 71.54% (357/499) patients had remarkably low total T cell counts, CD4^+^ and CD8^+^ T cell counts, respectively. Among milder disease patients in the Non-ICU group, the median value of total T cells, CD4^+^ and CD8^+^ T cell counts were 652, 342, and 208, respectively; the median value decreased to 261, 198, and 64.3, respectively, in the ICU group (Figure 1A). The counts of total T cells, CD4^+^, and CD8^+^ T cells were significantly lower in ICU patients than Non-ICU cases (Figure 1B). All these patients were further categorized into three groups based on age (<20 years old, 20–59 years and ≥60 years), and an age-dependent reduction of T cell numbers was observed in COVID-19 patients, with the lowest T cells numbers found in patients ≥60 years old (Figure 1C), suggesting a potential cause for increased susceptibility in elderly patients. It is worth noting that the age range of the ICU patients was 26–87 years [64.5 (53–70.75), Median (IQR)], suggesting that some young patients can become critically ill.

We next retrospectively reviewed T cell numbers in 212 cases from Non-ICU patients within one center (the General Hospital of Central Theater Command). The Non-ICU patients were further divided into four groups based on clinical outcomes. Among these patients, 151, 40 and 13 cases had mild/moderate, severe and critical disease, respectively, while 8 patient deaths occurred, in the perished group. Statistical analysis showed that T cell numbers including total T cells, CD4^+^ and CD8^+^ T cells in the severe and critical disease groups as well as the perished group were significantly lower than in the mild/moderate disease group. Most importantly, the numbers of total T cells, CD8^+^ T cells and CD4^+^ T cells in severe and perished groups were lower than 800, 300, or 400/μL, respectively (Figure 1D). This result suggests that there is a profound T cell loss in COVID-19 disease.

### Negative Correlations Between T Cell Numbers and Cytokines

The expression of angiotensin converting enzyme 2 (ACE2), the predicted receptor of SARS-CoV-2 viruses, is absent on T cells (11), suggesting that the depressed T counts in COVID-19 patients mentioned above (Figure 1) were likely not caused by direct infection of T cells. We therefore examined the concentrations of serum cytokines, including TNF-α, IFN-γ, IL-2, IL-4, IL-6, and IL-10, from these COVID-19 patients to explore the influence of cytokine signaling. We only found the levels of TNF-α, IL-6, and IL-10 were significantly increased in infected patients, and statistical analysis illustrated that their levels in ICU patients were significantly higher than in Non-ICU patients (Figure 2A). It is also worth noting that cytokine levels of some ICU patients were relatively normal, suggesting that a small minority of ICU patients were immunocompromised. The levels of IFN-γ, IL-2, and IL-4 showed no significant difference among different groups (Supplementary Figure 1A).

We next investigated the relationships between IL-10, IL-6, TNF-α, and T cell count within Non-ICU patients. Interestingly, the concentration of these three cytokines was negatively correlated with total T cell counts, CD4^+^ counts, and CD8^+^ counts, respectively (Figure 2B). We subsequently summarized the follow-up data of cytokine concentrations and T cell numbers in ten patients that were followed over the course of inpatient care. Interestingly, serum levels of IFN-γ, IL-10, IL-6, and TNF-α were significantly decreased in these patients in the decline (i.e., disease resolution) compared to the illness period, while counts of total T cells, CD4^+^, and CD8^+^ T cell subsets recovered during the decline period (Figure 2C). We also noted that serum levels of IL-2 and IL-4 had no difference between these two periods (Supplementary Figure 1B). The phenomena suggests that the decrease of T cells seen in COVID-19 patients may be the result of high serum concentration of TNF-α, IL-6, and IL-10 negatively regulating T cell survival or proliferation.

### Progressive T Cell Exhaustion With Severity of COVID-19 Disease

Beyond changing in numbers during the course of infection, T cells may display limited function during prolonged infection as a result of exhaustion, which has been associated with the expression of some immune-inhibitory factors including PD-1, Tim-3 on cell surface (12). We therefore examined whether T cells in COVID-19 patients have exhaustion phenotypes. FACS analysis illustrated that, in comparison to healthy controls, especially ICU patients with COVID-19 disease showed markedly higher percentages of PD-1^+^CD8^+^ and CD4^+^ T cells, with a trend to show higher PD-1 levels on PD-1^+^CD8^+^ or CD4^+^ T cells (Figures 3A,B), indicating that SARS-CoV-2 can drive T cell exhaustion in COVID-19 patients, particularly in those requiring ICU care.

Three patients were followed during inpatient care, and the expression of the exhaustion markers including PD-1 and Tim-3 on surface of T cells during disease progress was detected. FACS analysis showed that these patients had very low percentages of PD-1^+^ and Tim-3^+^ on CD8^+^ and CD4^+^ T cells in the prodromal stage of disease, however PD-1 and Tim-3 expression tended to progressively increase in CD8^+^ T cells during overtly symptomatic and ICU period disease stages (Figures 3C,D). Similarly, higher percentages of Tim-3^+^ cells were observed in CD4^+^ T cells from patients in the ICU stage, although PD-1 expression in CD4^+^ T cells was not obviously affected during disease progression (Figures 3C,D). Furthermore, PD-1 and Tim-3 protein expression tended to increase throughout the stages discussed above in PD-1^+^ or Tim-3^+^ CD8^+^ T cells (Supplementary Figure 1C). These results demonstrated that T cells are exhausted in COVID-19 patients during SARS-CoV-2 infection.

## Discussion

T cells play a vital role in viral clearance, with CD8^+^ cytotoxic T cells (CTLs) capable of secreting an array of molecules such as perforin, granzymes, and IFN-γ to eradicate viruses from the host (13). At the same time, CD4^+^ helper T cells (Th) can assist cytotoxic T cells and B cells and enhance their ability to clear pathogen (14). However, persistent stimulation by the virus may induce T cell exhaustion, leading to loss of cytokine production and reduced function (15, 16). Earlier studies have been unclear regarding the numbers and function of T cells in COVID-19 patients, albeit with suggestions of depressed lymphocyte counts (4, 6). In this report, we retrospectively reviewed the numbers of total T cells, CD4^+^, CD8^+^ T cell subsets in a total of 499 COVID-19 patients. In Non-ICU patients, we found that over 70.56% cases had a decrease in total, CD4^+^ and CD8^+^ T cells. However, in the ICU group, a total of 95% (19/20) patients showed a decrease in both total T cells and CD4^+^ T cells, and most importantly, all of the patients displayed decreases in CD8^+^ T cells. We also analyzed Non-ICU patients in greater detail, and found that urgent intervention may be necessary to preempt the development of severe symptoms in patients with low T cell counts.

Cytokine storm is a phenomenon of excessive inflammatory reaction in which cytokines are rapidly produced in large amount in response to microbial infection. This phenomenon has been considered an important contributor to acute respiratory distress syndrome (ARDS) and multiple organ dysfunction syndrome (MODS) (17, 18). It has been also implicated in the setting of respiratory viral infections, such as SARS in 2002, avian H5N1 influenza virus infection in 2005 and H7N9 infection in 2013 (19–22). Huang et al. showed that the levels of IL-2, IL-7, IL-10, TNF-α, G-CSF, IP-10, MCP-1, and MIP-1A were significantly higher in COVID-19 patients (4). Consistent with this report, here we found that the secretion of cytokines including TNF-α, IL-6, and IL-10 was increased in COVID-19 patients. Interestingly, the numbers of total T cells, CD4^+^ T and CD8^+^ T cells are negatively correlated to levels of TNF-α, IL-6, and IL-10, respectively (Figure 2B), suggesting these cytokines may be involved in the decrease of T cells detected in COVID-19.

TNF-α is a pro-inflammatory cytokine which can promote T cell apoptosis *via* interacting with its receptor, TNFR1, which expression is increased in aged T cells (23, 24). Our current analysis demonstrated that patients over 60 years old have lower T cell numbers, indicating that TNF-α might be directly involved in inducing T cell loss in these patients. IL-6, when promptly and transiently produced in response to infections and tissue injuries, contributes to host defense through the stimulation of acute phase responses or immune reactions. Dysregulated and continual synthesis of IL-6 has been shown to play a pathological role in chronic inflammation and infection (25, 26). Tocilizumab, a humanized anti-IL-6 receptor antibody, has been developed and approved for the treatment of rheumatoid arthritis (RA) and juvenile idiopathic arthritis (27, 28). Moreover, tocilizumab has been shown to be effective against cytokine release syndrome resulting from CAR-T cell infusion against B cell acute lymphoblastic leukemia (29). Whether tocilizumab can restore T cell counts in COVID-19 patients by suppressing IL-6 signaling remains uninvestigated.

The source of these cytokines during COVID-19 disease remains an open interesting issue. While previous studies have validated that the secretion of cytokines, including IL-6, IL-10, and TNF-α, mostly derives from T cells, macrophages and monocytes etc. (30, 31), based on our (inverse correlation) results, we suggest that the secretion of these cytokines does not originate from T cells. However, the cytokine storm in turn may promote apoptosis or necrosis of T cells, and consequently leads to their reduction. Our previous work demonstrated that monocytes and macrophages can produce pro-inflammatory cytokine during murine hepatitis virus strain-3 infection (32, 33), yet whether SARS-CoV-2 also triggers cytokine release from monocytes and macrophages in COVID-19 patients needs further investigation and current work around this is in progress in our hospital.

T cell exhaustion is a state of T cell dysfunction that arises during many chronic infections and cancer. It is defined by poor effector function, sustained expression of inhibitory receptors, and a transcriptional state distinct from that of functional effector or memory T cells (34). By FACS analysis, we found that both CD8^+^ T cells and CD4^+^ T cells have higher levels of PD-1 in virus infected patients, particularly in ICU patients (Figure 3). IL-10, an inhibitory cytokine, not only prevents T cell proliferation, but also can induce T cell exhaustion. Importantly, blocking IL-10 function has been shown to successfully prevent T cell exhaustion in animal models of chronic infection (35, 36). We demonstrate here that COVID-19 patients have very high levels of serum IL-10 following SARS-CoV-2 infection, while also displaying high levels of the PD-1 and Tim-3 exhaustion markers on their T cells, suggesting that IL-10 might be mechanistically responsible. The application of potent antiviral treatments to prevent the progression to T cell exhaustion in susceptible patients may thus be critical to their recovery. We have read with great interest the successful application of Remdesivir to cure a COVID-19 patient in the US, and clinical trials indicate that this drug may have significant potential as an antiviral (37, 38).

Taken together, we conclude that T cells are decreased and exhausted in patients with COVID-19. Cytokines such as IL-10, IL-6, and TNF-α might be involved in T cell reduction. Thus, new therapeutic measures are needed for treatment of COVID-19 ICU patients, and perhaps these are necessary even early on to preempt disease progression in higher-risk patients with low T cell counts.

## Data Availability Statement

The raw data supporting the conclusions of this article will be made available by the authors, without undue reservation.

## Ethics Statement

The studies involving human participants were reviewed and approved by National Health Commission of China and Ethics Commission of General Hospital of Central Theater Command ([2020]-004-1) and Hanyang Hospital (20200217). Written informed consent to participate in this study was provided by the participants' legal guardian/next of kin. Written informed consent was obtained from the individual(s), and minor(s)' legal guardian/next of kin, for the publication of any potentially identifiable images or data included in this article.

## Author Contributions

YW and YC were involved in the final development of the project and manuscript preparation. XC and YZ wrote the manuscript draft. ZY, CW, and ZF analyzed the data. BD, YL, YT, LN, LC, ML, YL, and GW performed most of experiments.

## Conflict of Interest

The authors declare that the research was conducted in the absence of any commercial or financial relationships that could be construed as a potential conflict of interest.

## Supplementary Material

Supplementary Figure 1 Cytokines and Exhaustion of T cells in COVID-19 patients. **(A)** Cytokine levels in different groups; **(B)** Dynamic profiles of cytokine levels in Non-ICU care patients; **(C)** Dynamic profile of PD-1 and TIM-3 expressions on T cells in 3 patients. NS, not significant.
