# Supplementary figures and images for: Reduction and Functional Exhaustion of T Cells in Patients With Coronavirus Disease 2019 (COVID-19)
Source: Front Immunol. 2020 May 1;11:827. doi: 10.3389/fimmu.2020.00827 (PMC7205903; doi:10.3389/fimmu.2020.00827)

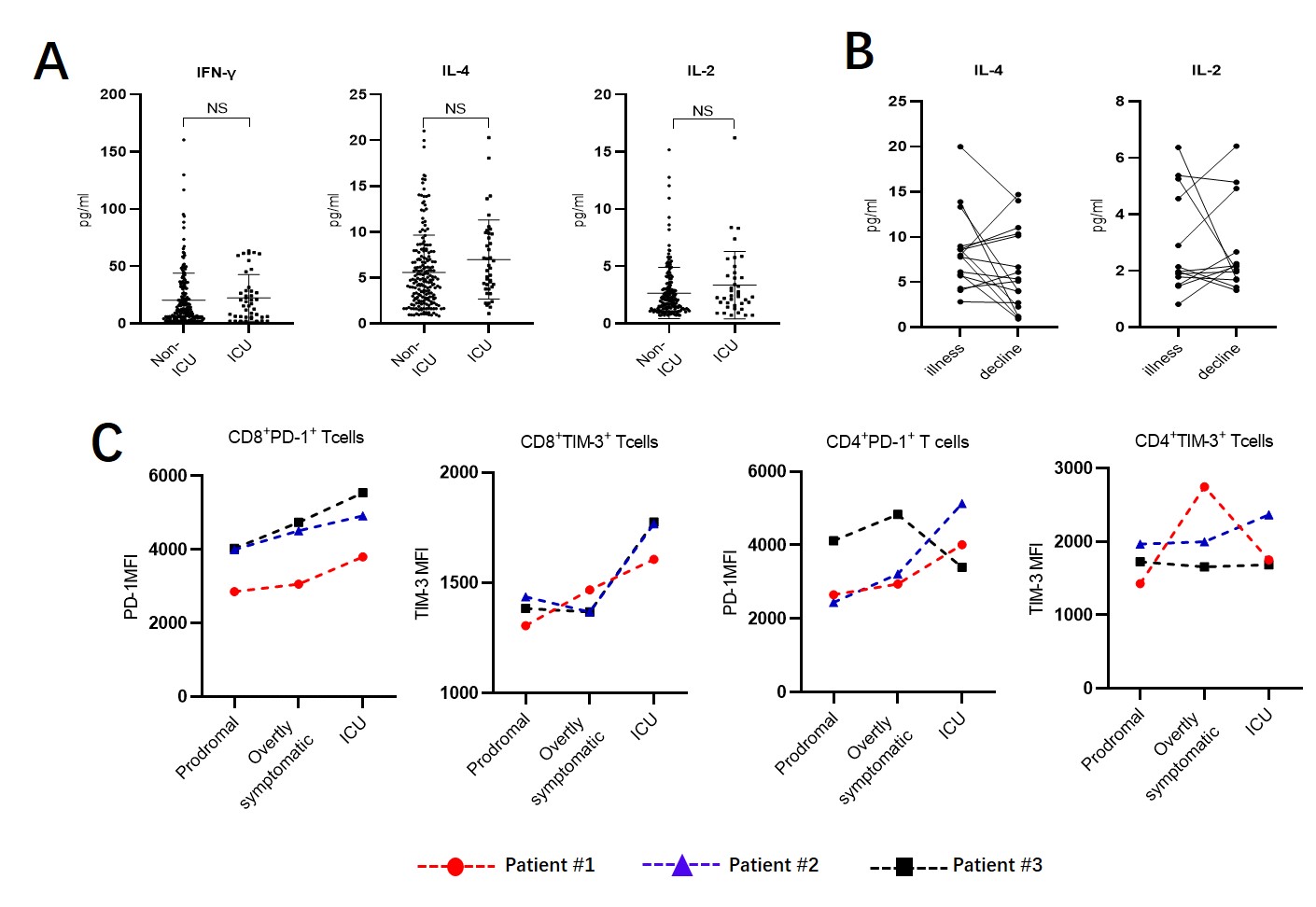

Supplement: Supplementary Figure 1 — Cytokines and Exhaustion of T cells in COVID-19 patients. (A) Cytokine levels in different groups; (B) Dynamic profiles of cytokine levels in Non-ICU care patients; (C) Dynamic profile of PD-1 and TIM-3 expressions on T cells in 3 patients. NS, not significant. [file Image_1.jpg]
